# The Valemee Visual System Helps Reduce Risk for Chronic Illness by Promoting Physical Fitness, Self‐Efficacy and Independence in Adults With Intellectual Disabilities

**DOI:** 10.1111/jar.70011

**Published:** 2025-01-13

**Authors:** Anthony Dujmovic‐Bračak, Alisa D. Blazek, Emily M. Post, Jacqueline D. Goodway, Carmen B. Swain

**Affiliations:** ^1^ The Ohio State University Columbus USA; ^2^ Franklin University Columbus USA; ^3^ Otterbein University Columbus USA

**Keywords:** intellectual disabilities, physical fitness, self‐efficacy, visual system

## Abstract

**Introduction:**

Sedentary behaviour among individuals with intellectual disabilities, driven by barriers such as limited access to adapted programs and low self‐efficacy, contributes to chronic health conditions. This study evaluates the effectiveness of the Valemee Visual System (VVS), a novel tool offering visual support and structured exercise programming, in improving physical fitness and promoting exercise independence in this population.

**Methods:**

A repeated measures design was employed with an 8‐week intervention involving 22 participants aged 22–44 with mild to moderate intellectual disabilities. The program utilised the VVS for total body fitness training.

**Results:**

Participants demonstrated significant improvements in grip strength, sit‐to‐stand performance, bench press capacity, 400‐m walk time, and flexibility. Self‐efficacy increased, and reduced reliance on prompts indicated greater exercise independence and autonomy.

**Conclusions:**

The VVS shows promise in enhancing physical fitness, accessibility, and adherence in adults with intellectual disabilities, supporting long‐term health and reducing chronic disease risk.

## 1 Introduction

### 1.1 Background/Rationale

Individuals with intellectual disabilities face higher risks of chronic health conditions and adverse health outcomes compared to the general population (Naaldenberg et al. 2015; Rimmer, Padalabalanarayanan, Malone, and Mehta 2017; Tracy and McDonald 2015), including increased susceptibility to gastrointestinal cancers (Cooke 1997; Liu et al. 2021), poorer physical fitness (Hutzler and Korsensky 2010), higher sedentary behaviour, and elevated rates of obesity‐related complications, such as cardiovascular disease and metabolic syndrome (Hsieh, Hilgenkamp, Murthy, et al. 2014; Lynch, McCarron, McCallion, and Burke 2022). Consequently, individuals with intellectual disabilities experience higher rates of premature mortality and morbidity (Vancampfort et al. 2020).

While exercise reduces disease risks and supports weight management, 58%–89% of individuals with ID fail to meet recommended physical activity (PA) guidelines (Hsieh et al. 2014). Effective interventions are critical for establishing and maintaining lifelong PA, but individuals with intellectual disabilities face barriers to exercise, including caregiver dependence, lack of adapted facilities, limited social support, transportation challenges, and financial constraints (Bossink, van der Putten, and Vlaskamp 2017; Rimmer et al. 2017). Compounding these extrinsic barriers are intrinsic factors, such as low self‐efficacy, limited experience with exercise programs, and low adherence (Gjestvang et al. 2020). Enhancing self‐efficacy and behavioural independence is vital to improving exercise participation and long‐term fitness (Jo, Rossow‐Kimball, and Lee 2018).

Although the benefits of exercise for individuals with intellectual disabilities are well‐documented, there remains a notable lack of adapted, evidence‐based programs that incorporate personalised tailoring, structured progression, and strategies to address barriers to exercise (Jeng, Chang, Liu, Hou, and Lin 2017; Obrusnikova, Firkin, and Farquhar 2022). Research indicates that adaptive exercise programs can mitigate fitness decline and lower the risk of chronic diseases (Calders, Elmahgoub, De Mettelinge, et al. 2011; Jo et al. 2018). However, ensuring the sustainability of exercise behaviour is critical for achieving and maintaining long‐term health benefits.

To address these challenges, this study evaluates the effectiveness of the Valemee Visual System (VVS), designed to improve physical fitness and exercise adherence in adults with intellectual disabilities. The VVS leverages visual input, which is a proven tool for teaching daily living skills (Spriggs, Mims, and Knight 2017), to promote PA and self‐efficacy. It includes portable, adaptive fitness equipment, and simple visual cues and activity options that encourage participant engagement and autonomy in exercise settings.

This study aimed to determine whether the VVS could enhance physical fitness and foster adherence to exercise programs in adults with intellectual disabilities, addressing critical gaps in the literature and providing a practical solution to improve health outcomes in this underserved population.

### 1.2 Objectives

This study aimed to evaluate the effectiveness of an 8‐week VVS‐guided exercise program in improving physical fitness (cardiovascular endurance, muscular strength, flexibility), behavioural independence, and exercise self‐efficacy in adults with mild to moderate intellectual disabilities. It was hypothesized that the VVS would enhance all three outcomes.

## 2 Method

### 2.1 Study Design

A repeated measures design was employed to account for variability in participants' IQs and physical abilities, with assessments conducted before and after the 8‐week intervention to evaluate within‐subject effects. Participants began with a consent and familiarisation session to acclimate to the exercise testing protocols, followed by a pretest within the same week. The guided exercise program consisted of two 35‐min sessions per week for eight weeks. Each session included a 5‐min cardiovascular warm‐up, 25 min of strength training targeting major muscle groups, such as the core, back, shoulders, arms, hamstrings, glutes, quadriceps, hip adductors and abductors, and chest, followed by stretching. Participants completed an average of five strengthening exercises per session, performing 2–3 sets of 10–15 repetitions (Pescatello, Arena, Riebe, and Thompson 2021). A posttest was conducted the week after completing the program.

### 2.2 Valemee Visual System

The VVS is a commercially available tool designed to foster exercise independence in individuals with intellectual disabilities by utilising visual aids (Spriggs et al. 2017) and customizable workout routines. It features a set of illustrated cards that depict whole‐body strength exercises, organised by major muscle groups and colour‐coded for ease of selection. Participants create personalised, full‐body workouts by choosing 1–2 cards from each category during each session, discarding cards as exercises are completed to provide a clear structure. The categories and exercises rotate weekly, offering a balanced, engaging program that adapts to the individual participant.

The VVS offers distinct benefits for both participants and practitioners. For participants, it promotes independence by providing structure, choice, and adaptability in exercise routines while maintaining consistency. Participants can personalise their workouts by selecting exercises, determining the order of execution, and making adaptations to meet their physical needs (Stancliffe, 2020). Practitioners, such as personal trainers and educators, are trained to implement a ‘faded feedback’ approach, which involves offering substantial guidance at the outset and gradually reducing support as participants develop greater autonomy and self‐reliance (Cengher, Martens, Farmer, and Miguel 2018). Additionally, the Valemee Tracker App facilitates real‐time tracking of participant progress, and the prompts provided during sessions. This integration of visual structure, personalised choice, and professional guidance fosters enhanced engagement, increased autonomy, and improved adherence to exercise routines among individuals with intellectual disabilities.

### 2.3 Setting

The 10‐week study included 8 weeks of training, with additional time for pretesting, familiarisation, and posttesting. All sessions took place at the Valemee Fitness Center, a specialised facility for individuals with physical, neurological, and developmental disabilities. Staffed by adaptive care professionals, the center provides personal training for individuals with intellectual disabilities, such as autism, Down syndrome, and cerebral palsy, and features traditional fitness equipment, including treadmills, free weights, benches, and weight machines.

### 2.4 Ethics

This study was approved by the university's Institutional Review Board (IRB) and adhered to the Declaration of Helsinki's ethical guidelines. Written informed consent was obtained from all legal guardians, and written assent from all participants, using simplified language and opportunities for questions to ensure comprehension.

Participant confidentiality was safeguarded by anonymizing data and assigning unique identifiers. Data were securely stored on encrypted, password‐protected servers accessible only to authorised personnel. Risks were minimised through supervised exercise sessions led by certified trainers, who provided individualised modifications based on participants' abilities. Regular breaks, hydration, and monitoring for fatigue or discomfort were incorporated.

Input from local advocacy groups and caregivers, including simplified consent, flexible communication, shorter sessions and familiar environments, shaped the study design to ensure inclusivity and respect for participants' needs. By collaborating with local advocacy groups and caregivers, the study design and implementation were informed by the unique needs of this population, ensuring inclusivity and respect for participants.

### 2.5 Participants

Participants were recruited via mass emails and phone calls through metro‐area organisations supporting adults with intellectual disabilities. Recruitment materials included a plain‐language letter for caregivers and facility personnel, along with an easy‐to‐read flier with visuals for display.

Eligibility was determined using the Physical Activity Readiness Questionnaire (PAR‐Q+) (Warburton, Jamnik, Bredin, Shephard, and Gledhill 2023). Participants were aged 22–44, had mild to moderate intellectual disability (IQ 35–70, assessed using the Kaufman Brief Intelligence Test (Bain and Gray 2008; Raggio, Scattone, and May 2010)), and no formal exercise programming or experience at Valemee Fitness within the past six months.

#### 2.5.1 Exclusion Criteria

Participants aged 18–21 were excluded due to the potential confounding effects of high school special education services, which in Ohio end after age 21. Adults over 44 were excluded due to the elevated cardiovascular disease risks (Liguori, Feito, Fountaine, and Roy 2022).

### 2.6 Variables

Three major dependent variables were examined: (1) physical fitness, (2) behavioural exercise independence, and (3) exercise self‐efficacy. A summary of the variables measured and the timing of collection during this study is presented in Table 1, and descriptions of the measures are described below.

**TABLE 1:** Variables and measures/instruments collected.

Variable	Measurement instrument	Collection of measure timing
Intelligence quotient	Kaufman brief intelligence test—2	Pretest
Age	Verbal intake	Pretest
Weight	Commercial scale	Pre & Posttest
Height	Stadiometer	Pre & Posttest
Sex	Verbal intake	Pretest
Race	Verbal intake	Pretest
Household status	Verbal intake	Pretest
Grip strength	Hand grip dynamometer test	Pre & Posttest
Lower body muscular endurance	30 Second sit to stand	Pre & Posttest
Upper body strength	6 Rep max bench press	Pre & Posttest
Cardiovascular fitness	400 Meter walk test	Pre & Posttest
Flexibility	Sit & reach	Pre & Posttest
Self‐efficacy for exercise	Self‐efficacy survey for exercise behaviours	Pre & Posttest

*Note:* It presents the variables and the measures/instruments used to collect data in the study.

### 2.7 Data Sources

#### 2.7.1 Physical Fitness Variables

Physical fitness was assessed through multiple measures. Upper limb strength was evaluated using a 6‐repetition maximum (6RM) bench press, a safer alternative to the standard 1RM testing for this population (Liguori et al. 2022). Grip strength, a reliable predictor of overall health and mortality, was measured with a handgrip dynamometer, with the best of two attempts per hand recorded (Carmelli, Imam, Levi, and Merrick 2013; Cuesta‐Vargas and Hilgenkamp 2015). Lower limb endurance was tested using a 30‐s sit‐to‐stand test (30 SSTS), where participants completed as many successful stands as possible without using their arms (McAllister and Palombaro, 2020). Cardiorespiratory fitness was assessed with a 400‐m walk test (400 MWT), with the time needed to complete 20 laps between cones recorded and breaks permitted but included in the timing (Focht et al. 2019). Flexibility was measured using the sit‐and‐reach test (S&R), with the furthest reach recorded across two attempts to evaluate spinal, lower back, and hamstring flexibility (Jo et al. 2018).

#### 2.7.2 Exercise Independence Variables

Exercise independence was evaluated by tracking trainer prompts delivered during sessions using the Valemee Tracker App. Prompts included verbal, visual, gestural, tactile, and physical guidance, provided when participants struggled with exercise execution, focus, or recalling steps. The app also recorded additional data, such as mood and behaviour, though not all were analysed in this study.

#### 2.7.3 Exercise Self‐Efficacy Variables

Exercise self‐efficacy was measured using the Self‐Efficacy Survey for Exercise Behaviours (Sallis, Pinski, Grossman, Patterson, and Nader 1988), administered to caregivers to gauge their perception of the participant's confidence in sustaining exercise behaviours. The survey included 12 items ranked on a Likert scale from 1 (I know I cannot) to 5 (I know I can), with scores averaged into two categories: “Sticking to It” and “Making Time for Exercise.” This approach mitigated comprehension and response biases common in the study population (Bradshaw, 2001; Finlay and Lyons 2002).

### 2.8 Controlling for Potential Bias

A familiarisation session before the pretest minimised learning effects, ensuring that pretest performance reflected participants' actual strength, endurance, or flexibility rather than familiarity with the tests (Oppewal, Hilgenkamp, Van Wijck, and Evenhuis, 2013). A single researcher conducted all pretest and posttest measures to eliminate inter‐rater reliability errors. Standardised instructions and consistent demonstrations were provided for all exercise tests and programming to maintain internal validity.

To control for prior training bias, participants with formal exercise experience within the past six months or prior exposure to Valemee Fitness were excluded, ensuring that all participants were novice exercisers. Encouragement during sessions was limited to essential prompts, such as “Let's move to the next station,” to avoid influencing participants' independence, with all prompts recorded in the VVS App.

### 2.9 Study Size

A power calculation using Python (version 3.6.9, statistical package 0.14.0) determined that 14 participants were needed to achieve 80% power with an effect size of 0.8 and an alpha of 0.05, which was suitable for exercise testing studies (Abt et al. 2020) based on Cohen's guidelines. To account for a 20% attrition rate and ineligibility following consent (e.g., age, IQ, PA readiness), 11 additional participants were added, resulting in a target enrollment of 25 participants.

### 2.10 Statistical Method

Descriptive statistics (mean, standard deviation, percentages) were reported for physical fitness, self‐efficacy, and prompt counts during fitness programming. Changes from pretest to posttest were analysed using paired sample t‐tests with a significance level of *p* = 0.05, and effect sizes (ES) calculated using Cohen's d (categorised as low < 0.2, moderate 0.2–0.8, and large > 0.8). Analyses were performed in R Statistical Software (version 4.2.2).

To assess associations between IQ levels (mild vs. moderate intellectual disability) and outcomes, change scores (posttest minus pretest) were computed, and two‐sample t‐tests were used to compare progress between groups.

Prompting trends over time were analysed using a log‐linear mixed model, with “time” (week number) as a fixed effect and “participant” as a random effect, addressing skewed data due to outliers. The geometric mean was used to account for variability, calculated as 𝑥̃ = exp. (1/𝑛 ∑ log(𝑥𝑖)).

## 3 Results

### 3.1 Participants

Of the 25 recruited participants, 96% adhered to the exercise program, and one dropped out due to time constraints. Two additional participants were excluded from the analysis due to IQ scores outside the mild to moderate intellectual disability range, leaving data from 22 participants (14 men and 8 women) for analysis.

### 3.2 Descriptive Data

Participant characteristics are detailed in Table 2. No significant changes in weight (81.81 ± 22.55 kg) or BMI (30.55 ± 7.18) were observed (paired *t*‐test). Of the 22 participants, 19 completed all 16 sessions, one completed 15, and two completed 14. Missing sessions resulted in lost prompting data, and analyses excluded these sessions.

**TABLE 2:** Participant demographics.

Demographic	Number (%)
IQ category	8 (36.4%) Mild 14 (63.6%) Moderate
Sex	8 (36.4%) Female 14 (63.6%s) Male
Race	3 (13.6%) African American 1 (4.5%) Asian 18 (81.8%) White
Household status	21 (95.5%) At home with family 1 (4.5%) Independent living

Demographic (range)	Mean (±SD)
Age (22–38 years)	26.91 (4.77)
Height (1.44–1.89 m)	1.63 (0.14)
Weight (51.45–122.02 kg; pre)	81.81 (22.55)
Weight (48.89–131.37 kg; post)	82.33 (23.34)
BMI (21.4–45.4 pre)	30.55 (7.18)
BMI (21.0–45.1 post)	30.74 (7.39)

*Note:* The demographic characteristics of the study participants.

## 4 Key Results

### 4.1 Physical Fitness Improvements

**FIGURE 1:**
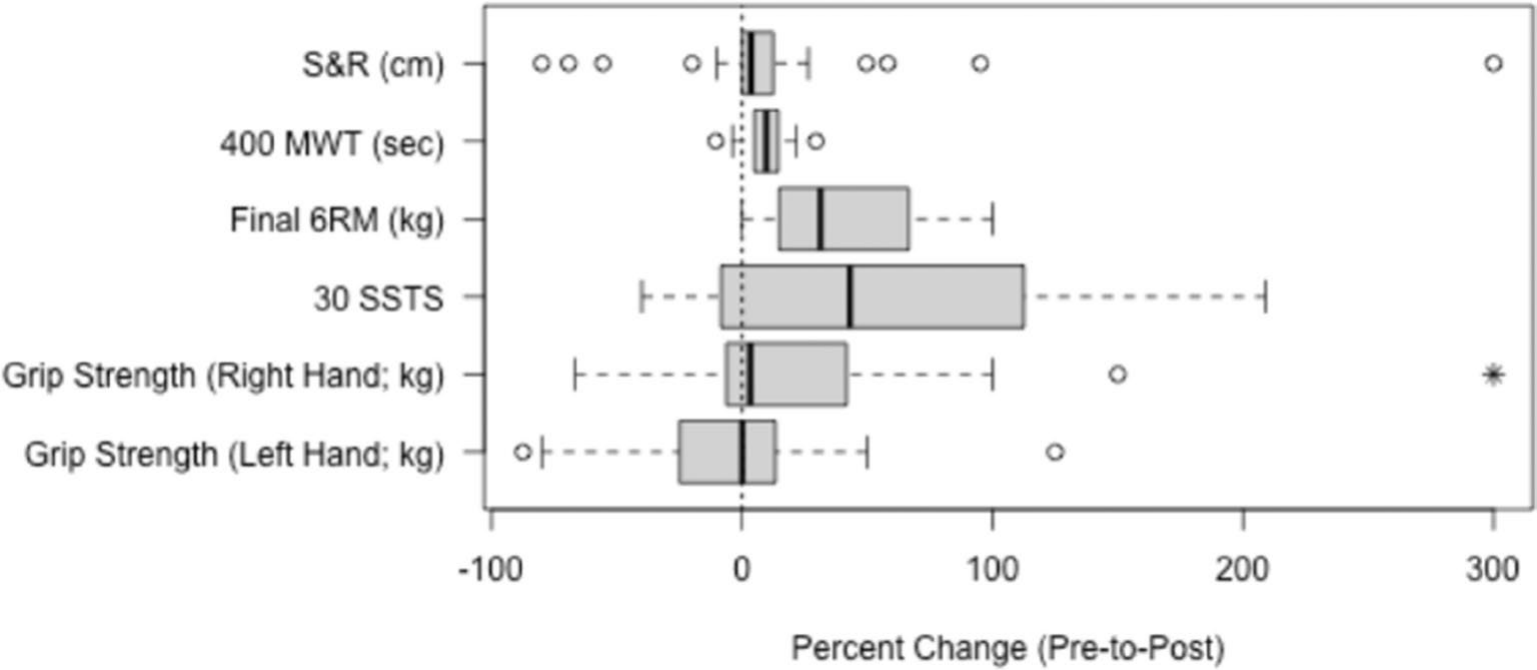
Percent change from baseline to posttest for physical fitness outcomes. This figure illustrates the percent change in physical fitness outcomes from baseline to posttest following the intervention. All physical fitness measures showed statistically significant improvements, except for left‐hand grip strength, as indicated in Table 3. Physical fitness outcomes are reported in various units, including 30 SSTS, Number of sit to standing position completed in 30 s; cm, centimetres; kg kilograms; sec, seconds.

All fitness measures improved significantly post‐intervention, except for left‐hand grip strength (Table 3). Percent changes from baseline are illustrated in Figure 1.

**TABLE 3:** The VVS intervention improved physical fitness outcomes.

Test	Pre‐mean (±SD)	Post‐mean (SD±)	Effect Size: Cohen's *d*	*p*	% Progress
Grip strength (Left hand; kg)	13.22 (10.02)	12.90 (10.91)	−0.030	0.6845	−2.5%
Grip strength (Right hand; kg)	13.32 (10.21)	15.09 (10.68)	0.170	0.028*	11.7%
30 Second sit to stand	10.05 (3.05)	15.00 (7.58)	0.858	0.002*	33.0%
Final 6RM (kg)	27.74 (11.76)	37.43 (14.56)	0.732	< 0.001*	25.9%
400 MWT (sec)	459.2 (140.40)	414.9 (135.50)	0.322	< 0.001*	9.6%
S&R (cm)	26.50 (18.19)	29.05 (18.18)	0.1403	0.044*	8.8%

*Note:* It presents the physical fitness outcomes following the VVS intervention. Grip Strength (Right hand), 30 SSTS, Final 6RM, 400 MWT, and S&R all significantly improved at the α = 0.05 level. Physical fitness outcomes are reported in various units, including cm, centimetres; sec, seconds, number of stands from sitting position completed in 30 s, and kg, kilograms.

* *p* < 0.05.

### 4.2 Self‐Efficacy Improvements

**FIGURE 2:**
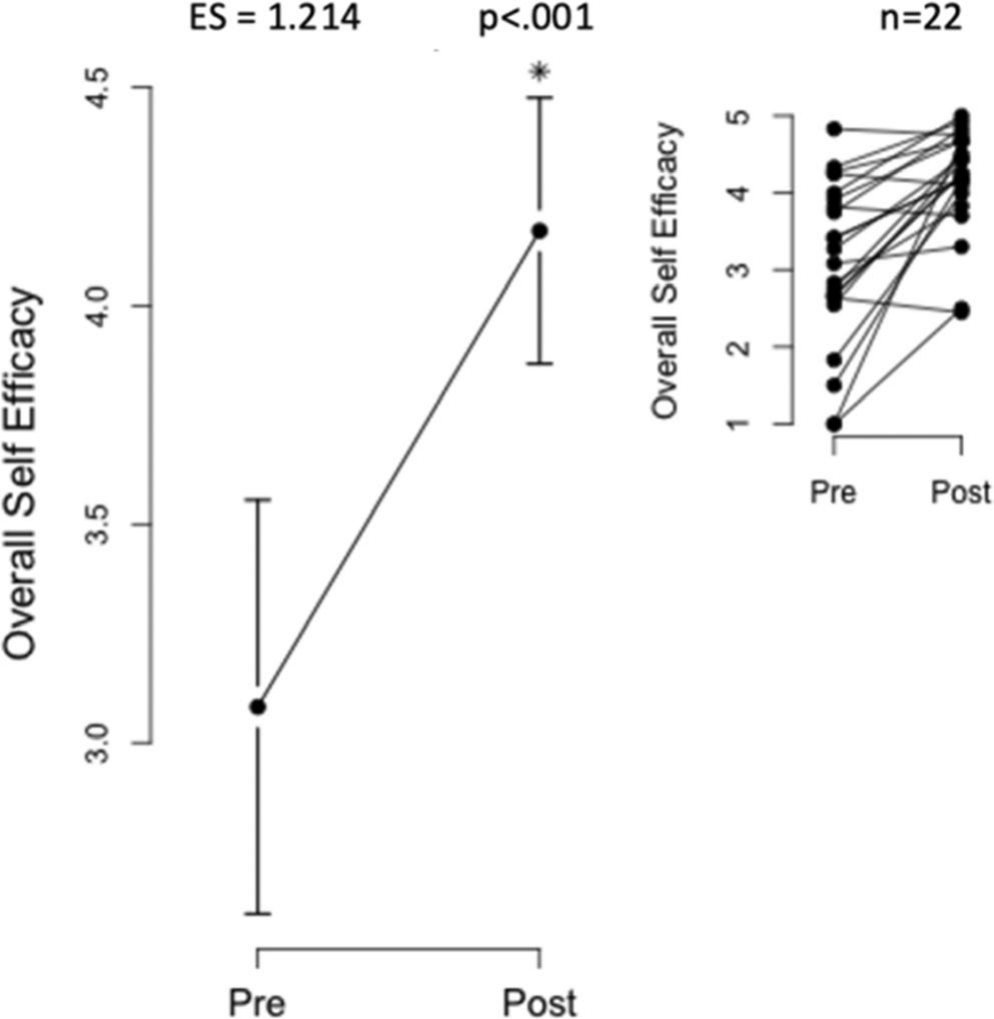
Significant increase in total self‐efficacy score. This figure highlights the substantial increase in the total self‐efficacy score following the intervention. Specifically, self‐efficacy for exercise demonstrated a marked improvement, with the total survey score showing an increase from 3.08 ± 1.07 to 4.17 ± 0.69. The statistical significance of this improvement was confirmed (*p* < 0.001*). The inset provides a visual representation of individual scores within this subset.

**FIGURE 3:**
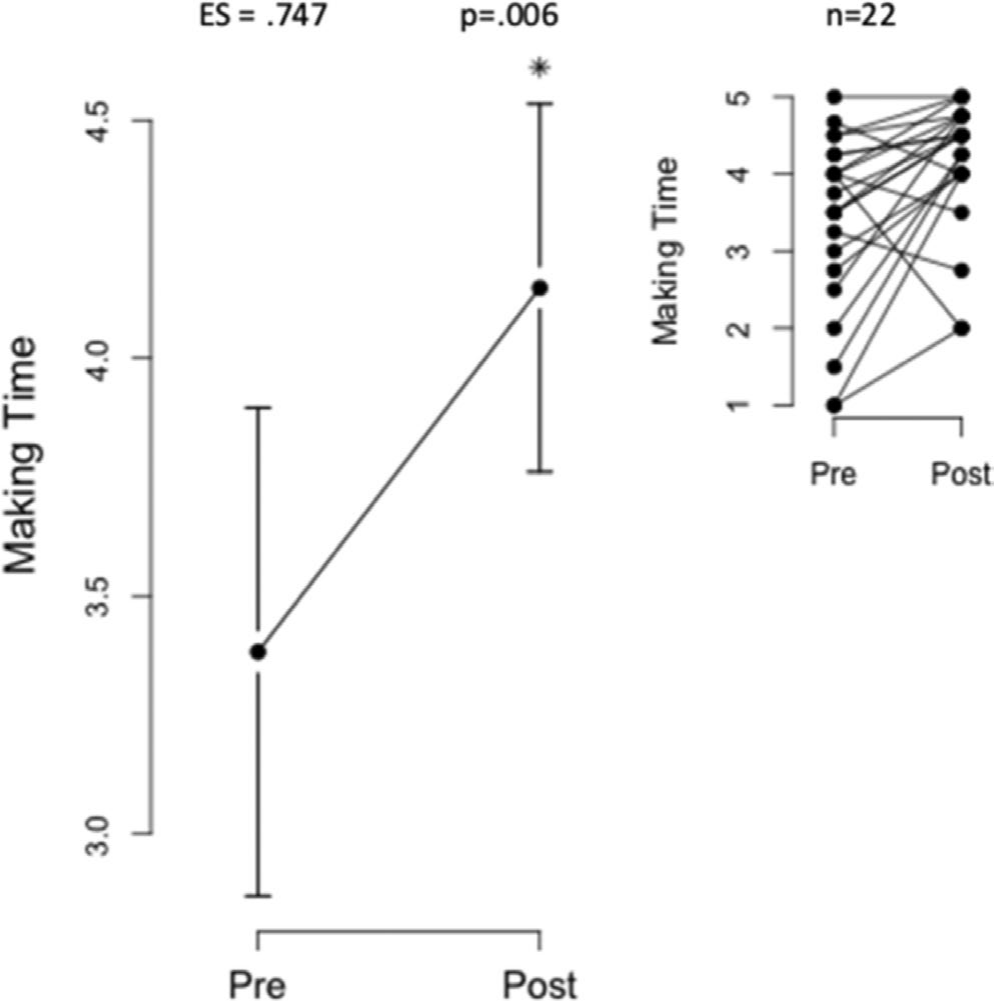
Significant increase in “making time” self‐efficacy subset score. This figure illustrates a noteworthy increase in self‐efficacy for exercise, specifically within the subset of “Making Time.” The data reveal that “Making Time” self‐efficacy scores improved from 3.38 ± 1.16 to 4.15 ± 0.87 after the intervention, with statistical significance (*p* = 0.006*). The inset provides a visual representation of individual scores within this subset.

**FIGURE 4:**
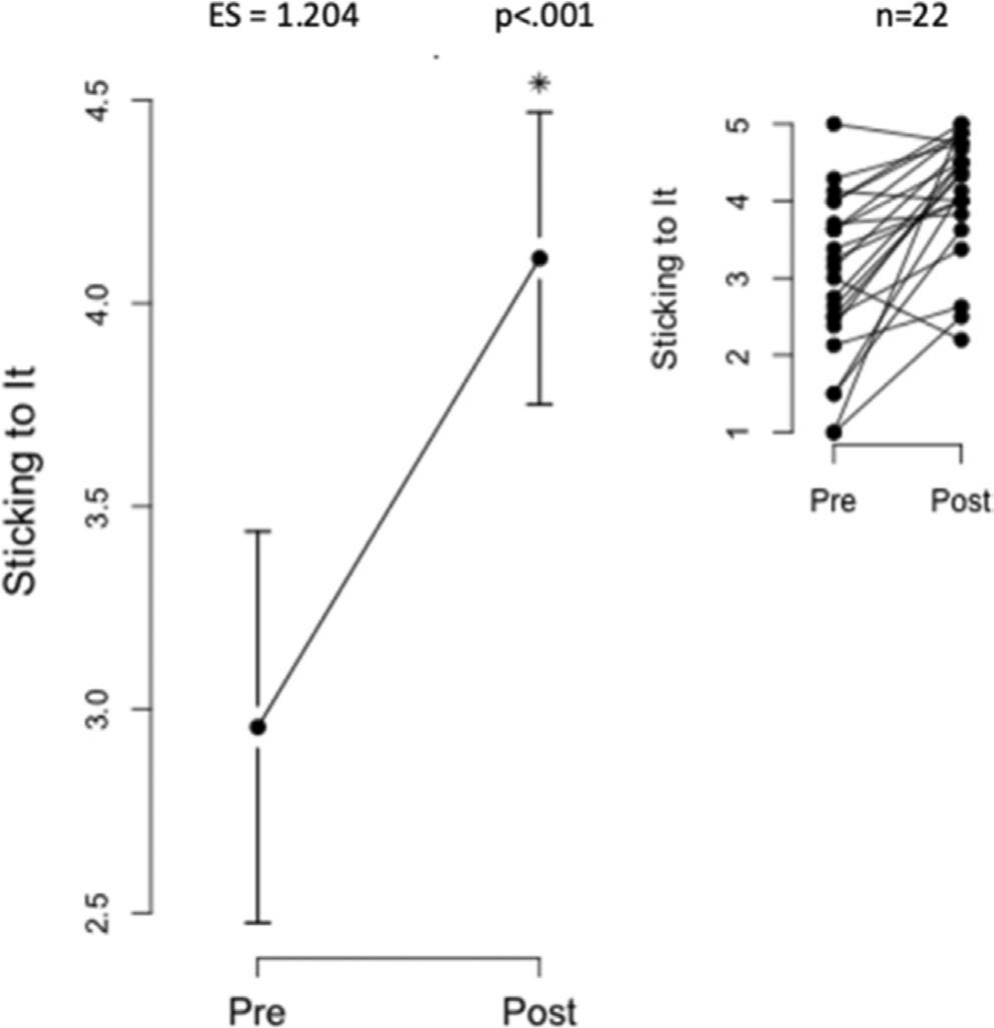
Significant Increase in “Sticking to It” Self‐Efficacy Subset Score. This figure highlights a substantial increase in self‐efficacy for exercise, specifically within the subset of “Sticking to It.” The data indicate that “Sticking to It” self‐efficacy scores improved from 2.96 ± 1.09 to 4.11 ± 0.81 following the intervention, with high statistical significance (*p* < 0.001*). The inset visually depicts individual scores within this subset for a more detailed insight into the changes observed.

Self‐efficacy scores increased significantly after the intervention, with total survey scores rising from 3.08 ± 1.07 to 4.17 ± 0.69. Subsets “Sticking to It” (2.96 ± 1.09–4.11 ± 0.81) and “Making Time” (3.38 ± 1.16–4.15 ± 0.87) also improved (Figures 2, 3, 4).

### 4.3 Exercise Independence Improvements

**FIGURE 5:**
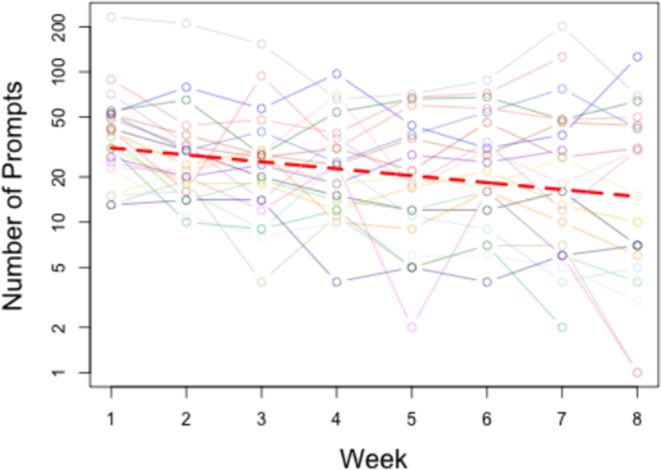
Total number of exercise prompts provided decreased over time. This figure illustrates the change in the total number of exercise prompts provided to participants throughout the study. The analysis was conducted by comparing data between Week 1 and Week 8, as well as examining week‐by‐week changes. Notably, there was a significant decrease in the total number of prompts delivered by study personnel to participants, with a remarkable reduction of 62.2% from Week 1 to Week 8. Figure 5 further demonstrates that while the prompt count exhibited variability among participants, there was a consistent linear decrease in prompts over time.

**FIGURE 6:**
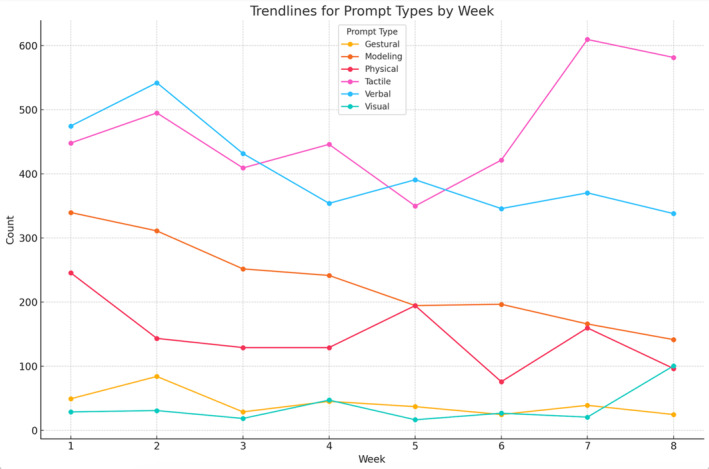
Trendlines for prompt types by week. This line graph illustrates the weekly trends for various prompt types (Visual, Verbal, Gestural, Modelling, Tactile, and Physical) used throughout the study. The x‐axis represents the intervention week (Week 1 to Week 8), and the y‐axis shows the total count of each prompt type recorded for that week. Each line corresponds to a specific prompt type, denoted by a unique marker and colour. Data points indicate the counts for a given week, providing insight into the frequency and variation of each prompt type over time. Error bars are not included, as the graph reflects aggregated counts rather than variability measures.

Prompt types delivered included tactile (35.3%), verbal (30.5%), modelling (17.3%), physical (11.0%), gestural (3.1%), and visual (2.7%). Total prompts decreased by 62.2% from Week 1 to Week 8, with variation across participants and a linear decline over time (Figure 5). Weekly declines are detailed in Table 4 and Figure 6.

**TABLE 4:** Change in total number of exercise prompts by week.

Week	1	2	3	4	5	6	7	8
Geometric mean number of prompts	36.14	26.13	22.81	22.36	17.23	21.74	18.10	13.67
Percent change from previous week	N/A	−28%	−13%	−2%	−23%	+26%	−17%	−24%

*Note:* The changes in the total number of exercise prompts required by participants each week during the intervention, using the geometric mean for better representation of relative changes.

## 5 Discussion

### 5.1 Key Results

This 8‐week exercise intervention utilising the VVS yielded significant improvements in physical fitness, self‐efficacy, and exercise autonomy among adults with intellectual disabilities. These findings hold profound significance, as physical inactivity is a major contributor to chronic disease prevalence. Recognising exercise as a proven therapy for both the prevention and management of chronic conditions (Booth, Roberts, and Laye 2012; Roberts and Barnard 2005), this intervention underscores the critical role of accessible and tailored fitness programs in promoting health and well‐being within this population.

The VVS addresses a critical gap by providing accessible and structured exercise programming for a population with limited PA opportunities post‐schooling. It enhances exercise adherence by offering simplicity, structure, and choice, improving both physical fitness and self‐efficacy—two key components for lifelong health maintenance (Broderick, Ryan, O'Donnell, and Hussey, 2019; Rimmer et al. 2017). Improvements in physical fitness, such as increased muscular strength and cardiorespiratory endurance, have clinically significant implications for conditions like sarcopenia, osteoporosis, and mobility disabilities (Kemmler & von Stengel, 2017; Liao, Tsauo, and Chen, 2023). For example, the participants' 400‐m walk test improved by a mean of 44.1 s, indicating the achievement of meaningful gains in mobility and function for daily living (Kwon et al. 2009).

Despite these advancements, participants' fitness levels remained below normative percentiles, likely attributable to poor baseline fitness and the time‐limited intervention period. These results highlight the need for ongoing, progressive exercise programming to achieve and sustain clinically significant improvements.

Behavioural outcomes, including enhanced self‐efficacy and independence, were closely tied to exercise adherence. The VVS leverages visual cues to promote autonomy, aligning with evidence supporting visual tools for teaching skills and fostering independence in individuals with intellectual disabilities (Spriggs et al. 2017). Long‐term use could help sustain exercise habits and prevent chronic conditions such as heart disease, diabetes, and cancer.

In conclusion, the VVS is a cost‐effective, portable tool that makes exercise accessible and sustainable for adults with intellectual disabilities. Its use in varied settings—schools, adult programs, fitness centers—can enhance physical fitness, self‐efficacy, and long‐term health outcomes in this underserved population.

## 6 Limitations and Recommendations for Future Research

Future studies should explore comprehensive lifestyle interventions that combine exercise with nutritional guidance to address multiple health aspects, particularly for individuals with overweight or obese BMI classifications. Such integrated approaches could enhance efforts to reduce chronic disease risk.

A key limitation of this study was the exclusion of adults with severe or profound intellectual disabilities, which limits generalizability. Including this population in future research would provide more inclusive insights and address their unique health challenges and barriers.

Self‐efficacy was measured via caregiver or representative reports rather than participant self‐assessments, a method supported by existing literature but potentially lacking in capturing participants' subjective experiences. Future research should explore adaptive self‐report tools to better assess self‐efficacy directly from participants. This study focused solely on exercise, with limited attention to dietary control. While exercise improved physical fitness and self‐efficacy, it did not result in significant weight changes, highlighting the need for future studies to target weight loss, especially given the participants' average BMI in the overweight to obese range. Longer intervention durations, larger sample sizes, and broader inclusion criteria could further enhance the generalizability and impact of future findings.

Lastly, understanding participants' intentions to maintain exercise beyond the intervention is critical. Future research should investigate factors influencing sustained engagement and develop strategies to support long‐term physical activity, fostering lasting health improvements.

## Author Contributions

**Anthony Dujmovic‐Bračak:** conceptualization (supporting); investigation (lead); formal analysis (supporting); writing – original draft (lead). **Alisa D. Blazek:** conceptualization (supporting); writing – original draft (supporting); supervision (supporting); writing – review and editing (equal). **Emily M. Post:** conceptualization (supporting); project administration (supporting); resources (supporting); methodology (supporting); writing – review and editing (supporting); **Jacqueline D. Goodway:** conceptualization (supporting); writing – original draft (supporting); writing – review and editing (supporting). **Carmen B. Swain:** conceptualization (lead); project administration (lead); methodology (lead); formal analysis (lead); supervision (lead); writing – original draft (supporting); writing – review and editing (equal).

## Ethics Statement

This study was approved by The Ohio State University Biomedical Research Ethics Committee.

## Conflicts of Interest

The authors declare no conflicts of interest.

## Data Availability

The data supporting the findings of this study are available from the corresponding author upon reasonable request.
